# Red LED Light Acts on the Mitochondrial Electron Chain of Donkey Sperm and Its Effects Depend on the Time of Exposure to Light

**DOI:** 10.3389/fcell.2020.588621

**Published:** 2020-12-07

**Authors:** Jaime Catalán, Marion Papas, Lina Trujillo-Rojas, Olga Blanco-Prieto, Sebastián Bonilla-Correal, Joan E. Rodríguez-Gil, Jordi Miró, Marc Yeste

**Affiliations:** ^1^Unit of Animal Reproduction, Department of Animal Medicine and Surgery, Faculty of Veterinary Medicine, Autonomous University of Barcelona, Bellaterra, Spain; ^2^Biotechnology of Animal and Human Reproduction (TechnoSperm), Institute of Food and Agricultural Technology, University of Girona, Girona, Spain; ^3^Unit of Cell Biology, Department of Biology, Faculty of Sciences, University of Girona, Girona, Spain

**Keywords:** sperm, red light stimulation, mitochondrial function, donkey, oligomycin A, FCCP

## Abstract

This work aimed to investigate how stimulation of donkey sperm with red LED light affects mitochondrial function. For this purpose, freshly diluted donkey semen was stimulated with red light for 1, 5, and 10 min, in the presence or absence of oligomycin A (Omy A), a specific inhibitor of mitochondrial ATP synthase, or FCCP, a specific disruptor of mitochondrial electron chain. The results obtained in the present study indicated that the effects of red LED light on fresh donkey sperm function are related to changes in mitochondria function. In effect, irradiation of donkey sperm resulted in an increase in mitochondrial membrane potential (MMP), the activity of cytochrome C oxidase and the rate of oxygen consumption. In addition, in the absence of oligomycin A and FCCP, light-stimulation augmented the average path velocity (VAP) and modified the structure of motile sperm subpopulations, increasing the fastest and most linear subpopulation. In contrast, the presence of either Omy A or FCCP abolished the aforementioned effects. Interestingly, our results also showed that the effects of red light depend on the exposure time applied, as indicated by the observed differences between irradiation protocols. In conclusion, our results suggest that exposing fresh donkey sperm to red light modulates the function of their mitochondria through affecting the activity of the electron chain. However, the extent of this effect depends on the irradiation pattern and does not exclude the existence of other mechanisms, such as those related to thermotaxis.

## Introduction

In recent years, the use and development of artificial insemination (AI) in equine species has grown considerably (Canisso et al., 2008; Crowe et al., 2008; Squires, 2009), and is currently being considered as the basis of modern equine reproduction worldwide (Pagl et al., 2006; Kowalczyk et al., 2019). However, while a significant number of previous studies have described and characterized the semen of domestic horses, studies on donkey sperm are scarce (Miró and Papas, 2018; Catalán et al., 2020a). In addition to this, at present, the most of European donkey breeds are in danger of extinction, which added to a mounting world demand for new donkey uses (milk, cosmetics or skin production, oncotherapy, silviculture, rural tourism…). For these reasons, the interest in developing studies to improve the reproductive performance in this species has increased (Miró et al., 2020). At this respect, it is worth noting that while both horses and donkeys are phylogenetically close species, they show important reproductive differences, and not only do their sperm vary in motility and morphology but also on how they interact with the female endometrium (Miró and Papas, 2018). Be that as it may, the fact that AI has been largely developed in the horse over the last years has fostered the application of those techniques in the donkey, which necessarily entails the need of developing methods, protocols or procedures aimed at optimizing sperm survival and fertilizing capacity, as in the horse (Loomis, 2006; Varner, 2016).

Irradiation of sperm with red light (laser and LED) has been demonstrated to jointly increase sperm motility and mitochondrial activity in sheep, cattle, pigs, donkeys and horses (Iaffaldano et al., 2016; Siqueira et al., 2016; Yeste et al., 2016; Catalán et al., 2020a,b,c). These effects seem, however, to rely upon the light-stimulation pattern, species and other factors, since while it has also been found to increase the *in vivo* fertilizing ability in pigs (Yeste et al., 2016; Blanco-Prieto et al., 2019), its use in separate farms around the world brings significant differences (Blanco-Prieto et al., 2019). In addition, the literature remains inconsistent on how irradiation affects sperm function and, at present, the mechanisms underlying that impact on mammalian spermatozoa remain largely unknown (reviewed in Yeste et al., 2018). Hence, further studies aimed at addressing the machinery that potentially guides the response of sperm to red light in separate species are warranted. In this context, the donkey could be an interesting model since previous studies have demonstrated that irradiation affects some sperm parameters in this species (Catalán et al., 2020a).

At present, three mechanisms have been suggested as being related to the response of sperm to red light (Yeste et al., 2018). The first hypothesizes that irradiation is linked to thermotaxis, via its interaction with specific members of the Transient Receptor Potential (TRP) family. While the TRP family is a highly heterogeneous class of plasma membrane receptors, only those belonging to vanilloid TRP (TRPV), ankyrin TRP (TRPA), and melastanin TRP (TRPM) subfamilies have been reported to be involved in the control of thermotaxis (Vriens et al., 2014). Remarkably, ion channels are crucial during sperm capacitation (Lishko et al., 2012; Singh and Rajender, 2014; Sun et al., 2017; Mundt et al., 2018), and TRPV4 has been linked to the thermotactic response both in mouse (Hamano et al., 2016) and human sperm (Mundt et al., 2018). A second potential mechanism envisages the participation of opsins, which are coupled to a G-protein (transducin) and also reside in the plasma membrane of mammalian sperm (Pérez-Cerezales et al., 2015). The different members of this family of proteins (melanopsin, encephalopsin, rhodopsin, and neuropsin) appear to work as thermosensitizers rather than light-sensitive intracellular proteins (photosensitizers), via canonical phospholipase C (PLC) and cyclic nucleotide pathways (cAMP/cGMP; Pérez-Cerezales et al., 2015). In effect, opsins, especially melanopsin and rhodopsin, are involved, along with the aforementioned TRPV proteins, in the sperm response to thermotaxis (Zheng, 2013; Pérez-Cerezales et al., 2015; Roy et al., 2020). Interestingly, a recent study has demonstrated that rhodopsin and melanopsin trigger a different signaling pathway but, unlike vision, both types of opsin coexist in the same sperm cells (Roy et al., 2020). The third hypothesis considers the role of endogenous photosensitizers, particularly cytochromes residing in the mitochondria (Yeste et al., 2018). It is widely known that cytochromes are an essential component of the mitochondrial electron chain and control oxidative phosphorylation, generation of reactive oxygen species and apoptosis (Ortega-Ferrusola et al., 2009). Cytochromes have a heme group, which not only accepts and donates electrons (Kessel, 1982; Pottier and Truscott, 1986), but is sensitive to light. However, not all cytochromes react against the same wavelengths (Lynch and Copeland, 1992). Thus, whereas cytochrome P450, which is also present in the endoplasmic reticulum of somatic cells, has its highest absorption peak at 450 nm, cytochrome C, which forms the complex IV of the mitochondrial electron chain, has two peaks at 610–630 nm and 660–680 nm (Lynch and Copeland, 1992). However, and despite unpublished data supporting the relevance of mitochondria in pig sperm (Blanco-Prieto et al., personal communication), one should not rule out that more than one of these hypothetical mechanisms could be involved in the sperm response to red light, as well as that the potential influence of light on the conformation of other proteins might also be implied in that response (Yeste et al., 2018).

Against this background, the objective of this study was to determine if the previously reported effects of light stimulation in donkey sperm (Catalán et al., 2020a) are related to changes caused by the action of light on mitochondrial function, since this is one of the hypothetical mechanisms of action. Our hypothesis is that the effects of irradiating donkey sperm with red-light are driven by changes in mitochondrial function. For this reason, freshly diluted donkey semen was irradiated with red light for 1, 5, and 10 min, in the presence/absence of Omy A, a specific inhibitor of the mitochondrial ATP synthase activity, or FCCP, which acts as a disruptor of the electron chain function. If mitochondria are involved in the sperm response to irradiation, utilizing these two effectors should affect the impact from red light. In addition, the use of both oligomycin A and FCCP should allow elucidating the exact part of the mitochondrial electron chain that is involved in the sperm response to light, and should also contribute to shed light onto the different effects between species.

## Materials and Methods

### Animals, Semen Samples, and Ethics

Eight ejaculates, each coming from a separate jackass, were used. Animals were allocated to individual paddocks at the Equine Reproduction Service, Autonomous University of Barcelona (Bellaterra, Cerdanyola del Vallès, Spain). This is an EU-approved semen collection center (Authorization code: ES09RS01E) that operates under strict protocols of animal welfare and health control. Jackasses were adult, healthy and of proven fertility, and were fed grain forage, straw and hay, with water being provided *ad libitum*.

Ejaculates were collected through a Hannover artificial vagina (Minitüb GmbH, Tiefenbach, Germany) and an in-line nylon mesh filter to remove the gel fraction. Upon collection, gel-free semen was diluted 1:5 (v:v) with Kenney extender (Kenney et al., 1975), previously preheated to 37°C. Thereafter, a conventional spermiogram, based on total ejaculate volume, sperm concentration (Neubauer chamber, Paul Marienfeld GmbH, and Co. KG; Lauda-Königshofen, Germany), motility (Computer Assisted Semen Analysis, CASA), morphology (eosin-nigrosin staining) and plasma membrane integrity (SYBR14/PI), was performed. All samples were confirmed to be above the standard thresholds (≥70% of total motility and SYBR14^+^/PI^–^ sperm and ≥ 70% morphologically normal sperm).

All jackasses used in this study were semen donors and no manipulation to animals, apart from semen collection in the authorized center, was made. The animal study was reviewed and approved by the Ethics Committee for Animal and Human Experimentation (CEEAH) of our institution (Autonomous University of Barcelona; authorization code: CEEAH 1424).

### Experimental Design

Prior to light-stimulation, sperm concentration was adjusted to 30 × 10^6^ spermatozoa/mL with Kenney extender using a Neubauer chamber (Paul Marienfeld GmbH & Co. KG; Lauda-Königshofen, Germany). Following this, each semen sample was split into separate aliquots of 1.5 mL that were subjected to three red light irradiation protocols with an air-refrigerated red LED light system (PhastBlue^®^, IUL, S.L.; Barcelona, Spain; wavelength range: 620–630 nm; intensity per sample: 35.05 W/m^2^). These three protocols consisted of exposing sperm to red light for 1 min (P1), 5 min (P5) or 10 min (P10). In all cases, the temperature within the PhastBlue^®^ system was maintained at 20°C ± 0.1°C. The control consisted of 1.5 mL tubes kept at 20°C in the dark for 10 min. In addition to the aforementioned, semen samples were also exposed to the same three protocols (i.e., P1, P5, and P10) or non-exposed (control), in the presence of either 5 μM oligomycin A or 5 μM FCCP. In order to achieve a maximal effect of both Omy A and FCCP, these two molecules were added to semen samples 10 min prior to exposure to red light. Following exposure to red light, sperm motility was evaluated through a computer-assisted sperm analysis system (CASA); sperm membrane integrity, mitochondrial membrane potential (MMP), and intracellular ROS and calcium levels were determined through flow cytometry; O_2_ consumption rate; intracellular ATP levels and total cytochrome C oxidase (CCO) activity were also assessed. In the case of intracellular ATP levels and total CCO activity, samples were first centrifuged at 2,000 × *g* and 20°C for 30 s; the resulting pellets were immediately frozen by plunging them into liquid N_2_. Samples were stored at −80°C until use.

### Sperm Motility

As indicated above, sperm motility was evaluated using a CASA system (Integrated Sperm Analysis System V1.0; Proiser S.L.; Valencia, Spain). For this purpose, samples were incubated in a water bath at 38°C for 5 min prior to analysis. Five microliter of each sperm sample was placed onto a Makler chamber (Sefi Medical Instruments; Haifa, Israel) previously warmed at 38°C. Samples were observed under a 10 × negative phase-contrast objective (Olympus BX41 microscope; Olympus Corporation, Tokyo, Japan), and at least 1,000 sperm cells were counted per analysis. In each evaluation, percentages of total (TMOT) and progressively motile spermatozoa (PMOT) were recorded along with the following kinetic parameters: curvilinear velocity (VCL, μm/s), which is the mean path velocity of the sperm head along its actual trajectory; straight-line velocity (VSL, μm/s), which is the mean path velocity of the sperm head along a straight line from its first to its last position; average path velocity (VAP, μm/s), which is the mean velocity of the sperm head along its average trajectory; percentage of linearity (LIN,%), which is the quotient between VSL and VCL multiplied by 100; percentage of straightness (STR,%), which is the quotient between VSL and VAP multiplied by 100; percentage of oscillation (WOB,%), which is the quotient between VAP and VCL multiplied by 100; mean amplitude of lateral head displacement (ALH, μm), which is the mean value of the extreme side-to-side movement of the sperm head in each beat cycle; and frequency of head displacement (BCF, Hz), which is the frequency at which the actual sperm trajectory crosses the average path trajectory (Hz).

Settings of the CASA system were those recommended by the manufacturer: frames/s: 25 images captured per second; particle area > 4 and < 75 μm^2^; connectivity: 6; minimum number of images to calculate the ALH: 10. Cut-off values were VAP ≥ 10 μm/s for TMOT, and STR ≥ 75% for PMOT. In addition, individual kinematic parameters (VSL, VCL, VAP, LIN, STR, and BCF) were used to determine motile sperm subpopulations. Three replicates were evaluated before calculating the corresponding mean ± SD.

#### Flow Cytometry

Flow cytometry was used to determine plasma membrane integrity, MMP, and intracellular levels of superoxides, peroxides and calcium, following the recommendations set by the International Society for Advancement of Cytometry (ISAC; Lee et al., 2008). In all analyses, sperm concentration was previously adjusted to 1 × 10^6^ spermatozoa/mL in a final volume of 500 μL, and three technical replicates were evaluated. Samples were examined using a Cell Laboratory QuantaSC cytometer (Beckman Coulter, Fullerton, CA, United States), and the sheath flow rate was set at 4.17 μL/min. Electronic volume (EV) and side scatter (SS) were recorded in a log-linear mode (EV/SS dot plots) for 10,000 events per replicate. The analyzer threshold was adjusted on the EV channel to exclude subcellular debris (particle diameter < 7 μm) and cell aggregates (particle diameter > 12 μm). When required, compensation was used to minimize fluorescence spill-over into a different channel. Information on all events was collected in List-mode Data files (EV, SS, FL1, FL2, and FL3) and processed using the Cell Lab QuantaSC MPL Analysis Software (version 1.0; Beckman Coulter). In all assessments, data were corrected using the procedure described by Petrunkina et al. (2010), based on the percentage of debris particles (SYBR14^–^/PI^–^) determined through SYBR14/PI staining. Fluorochromes were purchased from Molecular Probes^®^ (Invitrogen^®^, Thermo Fisher Scientific, Waltham, MA, United States) and diluted with dimethyl sulfoxide (DMSO).

#### Plasma Membrane Integrity

Sperm membrane integrity was assessed using the LIVE/DEAD Sperm Viability Kit (SYBR14/PI; Molecular Probes, Thermo Fisher Scientific), according to the protocol described by Garner and Johnson (1995) and adapted to donkey spermatozoa. In brief, samples were first incubated with SYBR14 (final concentration: 100 nM) at 38°C for 10 min, and then with PI (final concentration: 12 μM) at 38°C for 5 min. Three sperm populations were distinguished: (i) viable spermatozoa emitting green fluorescence (SYBR14^+^/PI^–^), which appeared on the right side of the lower half of FL1/FL3 dot plots; (ii) non-viable spermatozoa emitting red fluorescence (SYBR14^–^/PI^+^), which appeared on the left side of the upper half of FL1/FL3 dot plots; and (iii) non-viable spermatozoa emitting both green and red fluorescence (SYBR14^+^/PI^+^), which appeared on the right side of the upper half of FL1/FL3 dot plots. Non-stained particles (SYBR14^–^/PI^–^), which appeared on the left side of the lower half of FL1/FL3 dot plots, had similar EV/SS distributions than spermatozoa and were considered as non-DNA, debris particles. Percentages of non-stained particles were used to correct the percentages of double-negative sperm populations in the other assessments. Spill-over of FL1- into FL3-channel was compensated (2.45%).

#### Mitochondrial Membrane Potential

Mitochondrial membrane potential was determined through incubation with JC1 (5,5′,6,6′-tetrachloro-1,1′,3,3′tetraethyl−benzimidazolylcarbocyanine iodide; final concentration: 0.5 μM) at 38°C for 30 min in the dark. At low MMP, JC1 remains as a monomer (JC1_m__on_) emitting green fluorescence, which is detected through FL1. At high MMP, JC1 forms aggregates (JC1_a__gg_) emitting orange fluorescence, which is collected through FL2. Three sperm populations were distinguished: (i) spermatozoa with green-stained mitochondria (low MMP); (ii) spermatozoa with heterogeneous mitochondria stained both green and orange in the same cell (intermediate MMP); and (iii) spermatozoa with orange-stained mitochondria (high MMP). Ratios between JC1_a__gg_ (FL2) and JC1_m__on_ (FL1) fluorescence for each of these sperm populations were also evaluated. Spill-over of FL1- into FL2-channel was compensated (68.50%). Percentages of debris particles found in SYBR14/PI staining (SYBR14^–^/PI^–^) were subtracted from those of spermatozoa with low MMP, and percentages of all sperm populations were recalculated.

#### Analysis of Intracellular ROS Levels: H_2_O_2_ and ⋅O_2_^–^

Intracellular ROS levels were determined through two oxidation sensitive fluorescent probes: 2′,7′-dichlorodihydrofluorescein diacetate (H_2_DCFDA) and hydroethidine (HE), which detect hydrogen peroxide (H_2_O_2_) and superoxide anion (⋅O_2_^–^), respectively (Murillo et al., 2007). Sperm were counterstained with PI (H_2_DCFDA) or YO-PRO-1 (HE), following a protocol modified from Guthrie and Welch (2006).

With regard to peroxides, spermatozoa were incubated with H_2_DCFDA (final concentration: 200 μM) and PI (final concentration: 12 μM) at room temperature for 30 min in the dark. H_2_DCFDA is a stable, cell-permeable, non-fluorescent probe that, in the presence of H_2_O_2_, is deesterified and converted into 2′,7′-dichlorofluorescein (DCF). Fluorescence of DCF and PI were measured through FL1 and FL3 detectors, respectively. Four sperm populations were distinguished: (i) viable spermatozoa with low levels of peroxides (DCF^–^/PI^–^); (ii) viable spermatozoa with high levels of peroxides (DCF^+^/PI^–^); (iii) non-viable spermatozoa with low levels of peroxides (DCF^–^/PI^+^); and (iv) non-viable spermatozoa with high levels of peroxides (DCF^+^/PI^+^). Percentages of debris particles found in SYBR14/PI staining (SYBR14^–^/PI^–^) were subtracted from those of viable spermatozoa with low levels of peroxides (DCF^–^/PI^–^), and percentages of all sperm populations were recalculated. Spill-over of FL1- into the FL3-channel was compensated (2.45%). Data are shown as corrected percentages of viable spermatozoa with high levels of peroxides (DCF^+^/PI^–^), and geometric mean of DCF^+^-fluorescence intensity in the DCF^+^/PI^–^ sperm population.

As far as superoxides are concerned, samples were incubated with HE (final concentration: 4 μM) and YO-PRO-1 (final concentration: 25 nM) at room temperature for 30 min in the dark (Guthrie and Welch, 2006). Hydroethidine diffuses freely through plasma membrane and converts into ethidium (E^+^) in the presence of superoxide anions (⋅O_2_^–^) (Zhao et al., 2003). Fluorescence of ethidium (E) was detected through FL3 and that of YO-PRO-1 was detected through FL1. Four sperm populations were distinguished: (i) viable spermatozoa with low levels of superoxides (E^–^/YO-PRO-1^–^); (ii) viable spermatozoa with high levels of superoxides (E^+^/YO-PRO-1^–^); (iii) non-viable spermatozoa with low levels of superoxides (E^–^/YO-PRO-1^+^); and (iv) non-viable spermatozoa with high levels of superoxides (E^+^/YO-PRO-1^+^). Percentages of debris particles found in SYBR14/PI staining (SYBR14^–^/PI^–^) were subtracted from those of viable spermatozoa with low levels of superoxides (E^–^/YO-PRO-1^–^), and percentages of all sperm populations were recalculated. Spill-over of FL3-into the FL1-channel was compensated (5.06%). Data are shown as corrected percentages of viable spermatozoa with high levels of superoxides (E^+^/YO-PRO-1^–^), and geometric mean of E^+^-fluorescence intensity in the E^+^/YO-PRO-1^–^ sperm population.

#### Intracellular Calcium Levels

Previous studies found that Fluo3 mainly stains mitochondrial calcium in pig sperm (Yeste et al., 2015). For this reason, we combined this fluorochrome with PI (Fluo3/PI), as described by Kadirvel et al. (2009). Four sperm populations were identified: (i) viable spermatozoa with low levels of intracellular calcium (Fluo3^–^/PI^–^); (ii) viable spermatozoa with high levels of intracellular calcium (Fluo3^+^/PI^–^); (iii) non-viable spermatozoa with low levels of intracellular calcium (Fluo3^–^/PI^+^); and (iv) non-viable spermatozoa with high levels of intracellular calcium (Fluo3^+^/PI^+^). FL3 spill-over into the FL1-channel (28.72%) and FL1 spill-over into the FL3-channel (2.45%) were compensated.

#### Determination of Intracellular ATP Levels

Intracellular ATP levels were determined following the protocol set by Chida et al. (2012). Immediately after light-stimulation, 1-mL semen aliquots were centrifuged at 17°C for 30 s; pellets were plunged into liquid N_2_, and frozen pellets were subsequently stored at −80°C for a maximum of 3 weeks. Pellets were then resuspended in 300 μL ice-cold 10 mM 2-[4-(2-hydroxyethyl)piperazin-1-yl]ethanesulfonic acid (HEPES) buffer containing 250 mM sucrose (pH was adjusted to 7.4), and subsequently sonicated (10 kHz, 20 pulses; Bandelin Sonopuls HD 2070; Bandelin Electronic GmbH and Co., Berlin, Germany). During this process, tubes were kept on ice to avoid specimen heating. Next, samples were centrifuged at 1,000× g and 4°C for 10 min, supernatants were harvested for further use and pellets were discarded. Twenty μL of supernatant was used to determine total protein content, and the remaining volume was mixed with 300 μL ice-cold 10% (v:v) trichloroacetic acid and held at 4°C for 20 s. Samples were subsequently centrifuged at 1,000 × *g* and 4°C for 30 s, and supernatants were carefully separated from the pellet and again centrifuged at 1,000 × *g* and 4°C for 10 min. Supernatants were mixed with two volumes of 1 M Tris-acetate buffer (pH = 7.75), and the resulting suspension was used to determine the ATP content using the Invitrogen^®^ ATP Determination Kit (Thermo Fisher Scientific, Waltham, United States; catalog number: A22066). Determinations of ATP content were carried out with an Infinite F200 fluorimeter (TECAN^®^), using 96-wells microplates for fluorescence-based assays (Invitrogen^®^). Data were normalized against the total protein content determined with the Bradford method (Bradford, 1976) using a commercial kit (Bio-Rad laboratories; Hercules, CA, United States).

#### Determination of O_2_ Consumption Rate

Determination of O_2_ consumption rate was performed through the SensorDish^®^ Reader (SDR) system (PreSens Gmbh; Regensburg, Germany). After light-stimulation, 1 mL of each sperm sample was transferred onto an Oxodish^®^ OD24 plate (24 wells/plate). Plates were sealed with Parafilm^®^, placed within the SDR system, and incubated at 37°C (controlled atmosphere) for 2 h. During that time, O_2_ concentration was recorded in each well at a rate of one reading/min. Data were exported to an Excel file and final O_2_ consumption rate was normalized against the total number of viable spermatozoa per sample, which was determined through flow cytometry (SYBR14^+^/PI^–^ spermatozoa) as described above.

#### Determination of Cytochrome C Oxidase Activity

The CCO activity was determined in mitochondria-enriched sperm fractions, as described in Mclean et al. (1993). Briefly, 1-mL sperm aliquots, previously irradiated with red light, were centrifuged at 1,000 × *g* and 17°C for 30 s. The resulting pellets were immediately plunged into liquid N_2_ and stored for 3 weeks. Pellets were resuspended in 500 μL ice-cold PBS and sonicated (10 kHz, 20 pulses; Bandelin Sonopuls HD 2070). Thereafter, 500 μL Percoll^®^ (concentration: 1.055 mg/mL in PBS) at 4°C was placed onto each sperm homogenate. Samples were centrifuged at 3,000 × *g* and 10°C for 45 min and the mitochondria-enriched fraction was carefully harvested with a micropipette and transferred into a new 1.5 mL tube. Samples were again centrifuged at 12,000 × *g* and 20°C for 2 min and the resulting pellets were resuspended in 100 μL PBS at 20°C. These mitochondria-enriched suspensions were split into two separate aliquots. The first one was used to determine CCO activity using a commercial kit (Cytochrome C Oxidase Assay Kit; Sigma-Aldrich; catalog number CYTOCOX1). Enzyme activity was normalized against the total protein content. Therefore, the other aliquot (10 μL) was used to determine total protein content through a commercial kit (Bio-Rad laboratories) based on the Bradford method (Bradford, 1976).

### Statistical Analysis

Statistical analyses were conducted using a statistical package (SPSS^®^ Ver. 25.0 for Windows; IBM Corp., Armonk, NY, United States). Data were first tested for normal distribution (Shapiro-Wilk test) and homogeneity of variances (Levene test) and, if required, they were transformed with arcsin √x. The effects of irradiating donkey with red light and the presence of oligomycin A/FCCP were tested on sperm motility parameters, percentages of spermatozoa with an intact plasma membrane (SYBR14^+^/PI^–^), spermatozoa with high and intermediate MMP, viable spermatozoa with high intracellular calcium levels (Fluo3^+^/PI^–^), viable spermatozoa with high superoxide levels (E^+^/YO-PRO-1^–^), and viable spermatozoa with high peroxide levels (DCF^+^/PI^–^); geometric mean fluorescence intensities (GMFI) of JC1_a__gg_, Fluo3^+^, E^+^ and DCF^+^; JC1_a__gg_/JC1_m__on_ GMFI-ratios; intracellular levels of ATP; O_2_ consumption rate; and cytochrome C oxidase activity were evaluated through two-way analysis of variance (ANOVA; factor 1: irradiation protocol; factor 2: presence/absence of Omy A or FCCP) followed by *post hoc* Sidak test for pair-wise comparisons.

Motile sperm subpopulations were determined through the protocol described in Luna et al. (2017). In brief, individual kinematic parameters (VCL, VSL, VAP, LIN, STR, WOB, ALH, and BCF) recorded for each sperm cell were used as independent variables in a Principal Component Analysis (PCA). Kinematic parameters were sorted into PCA components and the obtained matrix was subsequently rotated using the Varimax method with Kaiser normalization. As a result, each spermatozoon was assigned a regression score for each of the new PCA components and these values were subsequently used to run a two-step cluster analysis based on the log-likelihood distance and the Schwarz Bayesian Criterion. Four sperm subpopulations were identified and each individual spermatozoon was assigned to one of these subpopulations (SP1, SP2, SP3, or SP4). Following this, percentages of spermatozoa belonging to each subpopulation were calculated per sample and used to determine the effects of irradiation and Omy A/FCCP on the distribution of motile sperm subpopulations through two-way ANOVA followed by Sidak test.

In all analyses, the level of significance was set at *P* ≤ 0.05. Data are shown as mean ± standard deviation (SD), median and interquartile range (i.e., Q1 and Q3).

## Results

### Effects of Red Light Stimulation on Plasma Membrane Integrity in the Presence or Absence of Either Omy A or FCCP

**FIGURE 1:**
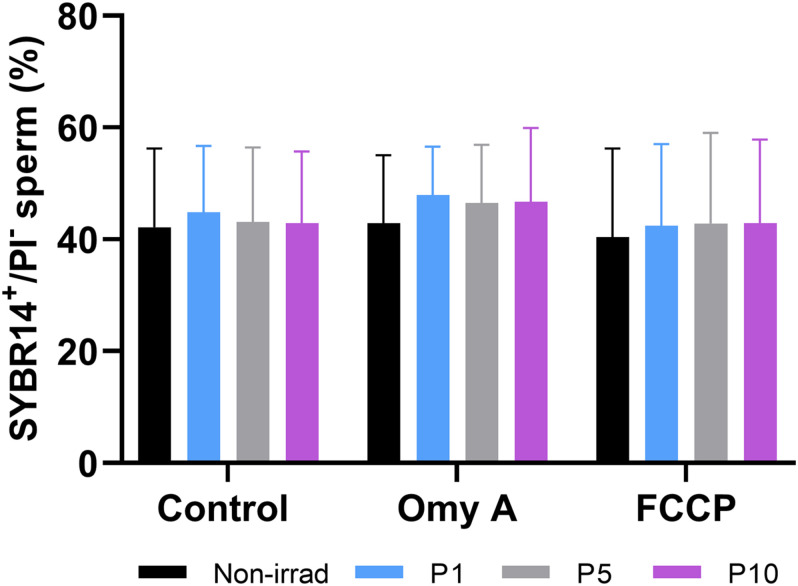
Effects of light-stimulation on the percentages of membrane-intact sperm (SYBR14^+^/PI^–^) in the presence/absence of Omy A or FCCP. No significant differences between non-irradiated and irradiated sperm in the presence/absence of oligomycin A or FCCP were observed, nor between samples in the presence of Omy A or FCCP and the treatment without the two disruptors for a given light-stimulation protocol (i.e., control, P1, P5, or P10). Data are shown as mean ± SD from eight separate experiments.

Irradiating donkey sperm with red light did not affect sperm membrane integrity, as no significant differences between the control and light-stimulation protocols were observed with regard to the percentages of membrane-intact spermatozoa (SYBR14^+^/PI^–^; Figure 1), neither did the presence of Omy A or FCCP alter that parameter.

### Effects of Red Light Stimulation on Sperm Motility in the Presence or Absence of Either Omy A or FCCP

**FIGURE 2:**
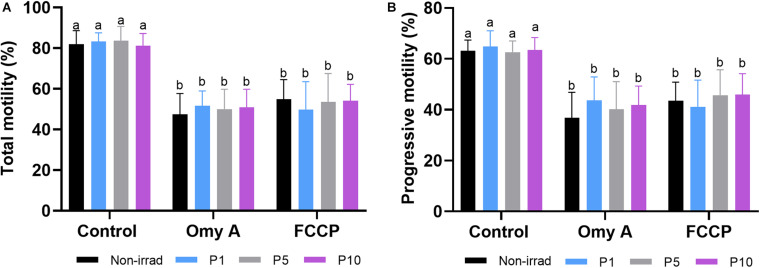
Effects of light-stimulation on the percentages of total **(A)** and progressive **(B)** sperm motility in the presence/absence of oligomycin A (Omy A) or FCCP. Different letters (*a b*) indicate significant differences (*P* < 0.05) between oligomycin A or FCCP with respect to the treatment without the two for a given protocol (i.e., control, P1, P5, or P10). No significant differences were found between the samples exposed to the different irradiation times with respect to non-irradiated sperm. Data are shown as mean ± SD from eight separate experiments.

As shown in Figure 2, percentages of TMOT (Figure 2A) and PMOT (Figure 2B) did not differ between non-irradiated and irradiated sperm samples, in the presence/absence of either Omy A or FCCP. Moreover, the presence of Omy A or FCCP, irrespective of irradiation/non-irradiation, led to a significant (*P* < 0.05) decrease in total (Figure 2A) and progressive (Figure 2B) sperm motility.

Regarding the effects of light-stimulation on sperm kinetic parameters (Table 1), irradiation for 5 min and 10 min significantly (*P* < 0.05) increased VAP, when Omy A and FCCP were absent. In contrast, the presence of Omy A or FCCP in non-irradiated samples led to a significant (*P* < 0.05) decrease in VCL, VSL, VAP, LIN, and WOB (Tables 1A,B). Moreover, treatments with Omy A and FCCP showed significantly (*P* < 0.05) higher values of BCF (Table 1C) and STR in non-irradiated and irradiated samples.

**TABLE 1A:** Kinetic parameters (VSL, VCL, and VAP) of donkey sperm in the control and the different light-stimulation patterns in the presence or absence of oligomycin A (Omy A) and FCCP.

Treatments		Kinetic parameters					
		VCL (μm/s)		VSL (μm/s)		VAP (μm/s)	
		Mean ± SD	Q1, median, Q3	Mean ± SD	Q1, median, Q3	Mean ± SD	Q1, median, Q3
Control	Control	130.7 ± 13.5^Aa^	110.0, 133.0, 147.5	102.1 ± 10.9^Aa^	89.0, 105.3, 111.6	117.6 ± 6, 9^Aa^	101.3, 121.0, 131.1
	Omy A	66.0 ± 8.7^Ab^	55.6, 64.7, 77.1	42.4 ± 8.7^Ab^	30.8, 44.2, 47.6	46.5 ± 6.0^Ab^	36.0, 49.1, 53.7
	FCCP	75.8 ± 9.8^Ab^	69.5, 75.9, 87.8	44.0 ± 9.5^Ab^	35.1, 46.4, 49.9	49.2 ± 6.9^Ab^	40.7, 52.2, 57.4
P1	Control	135.2 ± 15.6^Aa^	110.6, 137.4, 156.4	105.1 ± 13.3^Aa^	88.6, 107.3, 120.5	126.3 ± 9.9^Aa^	103.2, 129.0, 138.8
	Omy A	68.4 ± 8.1^Ab^	59.4, 66.6, 77.1	43.2 ± 7.4^Ab^	35.2, 46.9, 50.7	47.7 ± 5.5^Ab^	39.2, 50.4, 55.0
	FCCP	73.4 ± 11.8^Ab^	63.5, 70.1, 91.5	41.2 ± 10.6^Ab^	31.9, 45.7, 51.8	46.5 ± 8.9^Ab^	34.1, 50.8, 58.3
P5	Control	139.6 ± 11.6^Aa^	123.7, 141.5, 153.4	109.2 ± 8.9^Aa^	94.9, 108.2, 117.4	130.7 ± 6.2^Ba^	115.3, 131.8, 142.5
	Omy A	69.2 ± 10.8^Ab^	55.8, 65.9, 82.6	44.5 ± 10.6^Ab^	35.1, 46.0, 54.4	49.5 ± 5.5^Ab^	38.9, 51.1, 60.7
	FCCP	80.4 ± 12.3^Ab^	69.2, 81.8, 92.2	48.6 ± 11.5^Ab^	39.4, 49.6, 54.2	53.0 ± 6.4^Ab^	43.7, 54.8, 59.2
P10	Control	141.2 ± 10.7^Aa^	132.6, 142.2, 158.7	110.0 ± 11.6^Aa^	99.5.0, 111.2, 119.5	132.1 ± 6.4^Aa^	122.4, 133.9, 142.3
	Omy A	66.9 ± 8.1^Ab^	57.4, 66.2, 76.8	41.9 ± 7.5^Ab^	34.5, 42.8, 49.8	46.1 ± 5.5^Ab^	38.4, 47.3, 54.0
	FCCP	78.2 ± 11.7^Ab^	68.4, 76.9, 90.5	47.1 ± 10.6^Ab^	37.1, 49.1, 53.7	54.2 ± 6.2^Ab^	41.4, 53.7, 59.3

**Different letters (A,B) indicate significant differences (P < 0.05) between the control and the different light stimulation patterns used in the presence or absence of oligomycin A (Omy A) or FCCP. Different letters (a,b) indicate significant differences (P < 0.05) between Omy A or FCCP with respect to treatment without the two inhibitors for a given protocol (i.e., control, P1, P5, or P10). Data are shown as mean ± SD, median and interquartile range from eight separate experiments.**

**TABLE 1B:** Kinetic parameters (LIN, STR and WOB) of donkey sperm in the control and the different light-stimulation patterns in the presence or absence of oligomycin A (Omy A) and FCCP.

Treatments		Kinetic parameters					
		LIN (%)		STR (%)		WOB (%)	
		Mean ± SD	Q1, median, Q3	Mean ± SD	Q1, median, Q3	Mean ± SD	Q1, median, Q3
Control	Control	77.8 ± 3.4^Aa^	69.6, 79.7, 84.8	85.2 ± 2.1^Aa^	78.5.6, 87.1, 90.7	91.3 ± 3.6^Aa^	88.7, 91.1, 96.2
	Omy A	63.9 ± 7.7^Ab^	55.0, 64.6, 72.0	90.3 ± 2.4^Ab^	87.3, 91.7, 92.8	70.6 ± 8.0^Ab^	63.1, 70.4, 79.6
	FCCP	56.2 ± 7.5^Ab^	49.3, 59.2, 63.1	88.0 ± 2.9^Aa^	87.8, 90.4, 90.8	62.9 ± 7.9^Ab^	56.1, 65.2, 69.6
P1	Control	78.3 ± 2.6^Aa^	72.9, 77.8, 84.2	84.9 ± 1.8^Aa^	79.7, 85.2, 90.3	92.1 ± 2.8^Aa^	91.2, 91.7, 93.3
	Omy A	63.2 ± 6.9^Ab^	56.9, 62.0, 69.1	90.4 ± 1.4^Ab^	89.2, 90.5, 91.3	69.8 ± 7.9^Ab^	63.5, 68.9, 76.3
	FCCP	56.0 ± 7.8^Ab^	49.4, 54.7, 61.6	88.3 ± 2.2^Aa^	87.3, 88.5, 90.1	63.3 ± 8.3^Ab^	56.7, 61.2, 69.8
P5	Control	78.1 ± 3.2^Aa^	67.7, 81.1, 86.2	84.2 ± 2.2^Aa^	78.9, 86.8, 90.7	93.0 ± 3.6^Aa^	89.1, 91.4, 95.8
	Omy A	64.3 ± 6.2^Ab^	54.6, 67.5, 72.1	89.7 ± 2.4^Ab^	86.6, 89.8, 92.5	71.5 ± 8.6^Ab^	63.0, 74.1, 78.5
	FCCP	59.5 ± 9.7^Ab^	50.1, 56.9, 69.9	90.3 ± 1.4^Ab^	88.6, 91.1, 91.6	65.7 ± 10.3^Ab^	55.9, 63.0, 75.5
P10	Control	78.3 ± 3.5^Aa^	71.0, 77.9, 86.4	83.8 ± 2.7^Aa^	80.3, 84.8, 90.6	93.1 ± 4.8^Aa^	88.4, 91.9, 96.0
	Omy A	62.6 ± 8.3^Ab^	55.3, 60.1, 70.8	90.8 ± 1.6^Ab^	89.2, 90.9, 92.5	68.8 ± 8.7^Ab^	61.9, 65.3, 77.7
	FCCP	60.2 ± 10.0^Ab^	50.9, 58.7, 68.5	90.4 ± 1.1^Ab^	89.6, 90.3, 90.9	66.5 ± 10.4^Ab^	57.0, 65.1, 74.3

**Different letters (A,B) indicate significant differences (P < 0.05) between the control and the different light stimulation patterns used in the presence or absence of oligomycin A (Omy A) or FCCP. Different letters (a,b) indicate significant differences (P < 0.05) between Omy A or FCCP with respect to treatment without the two inhibitors for a given protocol (i.e., control, P1, P5, or P10). Data are shown as mean ± SD, median and interquartile range from eight separate experiments.**

**TABLE 1C:** Kinetic parameters (ALH and BCF) of donkey sperm in the control and the different light-stimulation patterns in the presence or absence of oligomycin A (Omy A) and FCCP.

TreatmentS		Kinetic parameters			

		ALH (μm)		BCF (Hz)	
		Mean ± SD	Q1, median, Q3	Mean ± SD	Q1, median, Q3
Control	Control	2.5 ± 0.7^Aa^	1.8, 2.6, 3.3	9.2 ± 1.0^Aa^	8.1, 9.0, 10.3
	Omy A	2.4 ± 0.3^Aa^	2.1, 2.4, 2.7	12.9 ± 1.0^Ab^	10.4, 12.6, 13.5
	FCCP	2.8 ± 0.3^Aa^	2.5, 2.9, 3.0	13.3 ± 0.7^Ab^	12.3, 13.3, 13.8
P1	Control	2.6 ± 0.4^Aa^	1.9, 2.5, 3.2	9.1 ± 1.1^Aa^	7.7, 8.9, 11.0
	Omy A	2.5 ± 0.2^Aa^	2.3, 2.5, 2.7	12.7 ± 0.8^Ab^	11.0, 12.5, 13.1
	FCCP	2.9 ± 0.4^Aa^	2.5, 2.8, 3.2	12.9 ± 0.9^Ab^	12.2, 12.7, 13.7
P5	Control	2.6 ± 0.6^Aa^	2.0, 2.5, 3.4	8.9 ± 1.0^Aa^	7.7, 8.8, 10.4
	Omy A	2.5 ± 0.4^Aa^	2.2, 2.4, 2.8	11.8 ± 0.9^Ab^	10.6, 12.0, 12.9
	FCCP	2.9 ± 0.3^Aa^	2.6, 3.0, 3.1	13.8 ± 1.0^Ab^	11.8, 13.6, 14.2
P10	Control	2.7 ± 0.6^Aa^	2.0, 2.8, 3.5	9.4 ± 0.9^Aa^	8.2, 9.4, 10.6
	Omy A	2.5 ± 0.3^Aa^	2.2, 2.4, 2.7	12.3 ± 0.9^Ab^	11.0, 12.4, 13.3
	FCCP	2.8 ± 0.4^Aa^	2.4, 2.8, 3.1	13.8 ± 0.8^Ab^	12.1, 13.7, 14.0

**Different letters (A,B) indicate significant differences (P < 0.05) between the control and the different light stimulation patterns used in the presence or absence of oligomycin A (Omy A) or FCCP. Different letters (a,b) indicate significant differences (P < 0.05) between oligomycin A or FCCP with respect to treatment without the two inhibitors for a given protocol (i.e., P1, P5, or P10). Data are shown as mean ± SD, median and interquartile range from eight separate experiments.**

**FIGURE 3:**
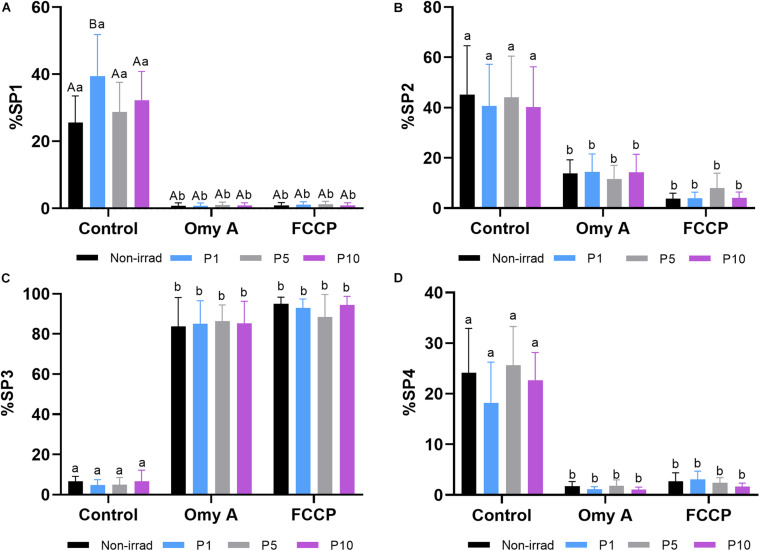
Effects of light stimulation on the structure of the motile sperm subpopulation in the presence/absence of oligomycin A (Omy A) or FCCP. **(A)** Subpopulation 1 (SP1, the fastest and most linear); **(B)** Subpopulation 2 (SP2 presented intermediate values, lower than SP1 and SP4, but more linear than SP4; **(C)** Subpopulation 3 (SP3, the slowest, but more linear than SP4); and **(D)** Subpopulation 4 (SP4 presented intermediate values, higher than SP2, but was the least linear). Different letters (*A,B*) indicate significant differences (*P* < 0.05) between non-irradiated samples and the different light stimulation patterns used in the presence or absence of oligomycin A (Omy A) or FCCP. Different letters *(a,b*) indicate significant differences (*P* < 0.05) between oligomycin A (Omy A) or FCCP with respect to the treatment without the two disruptors for a given protocol (i.e., control, P1, P5, or P10). Data are shown as mean ± SD from eight separate experiments.

Finally, as shown in Table 2, four different motile sperm subpopulations were identified in all treatments (SP1, SP2, SP3, and SP4). Interestingly, irradiation was found to affect the proportions of sperm belonging to each motile subpopulation. Light-stimulation of spermatozoa with red light for 1 min significantly increased (*P* < 0.05) the proportions of sperm belonging to SP1 (Figure 3A), which was the subpopulation that exhibited the highest kinematic parameters (Table 2). Regardless of whether samples were or not irradiated, the presence of Omy A or FCCP significantly (*P* < 0.05) decreased the proportions of sperm belonging to SP1, SP2, and SP4 (Figures 3A,B,D) and increased those of sperm belonging to SP3 (Figure 3C), which was the motile subpopulation that exhibited the lowest values in most kinematic parameters (Table 2).

**TABLE 2:** Descriptive parameters (mean ± SD, median and interquartile range) of the four sperm subpopulations (SP1, SP2, SP3, and SP4) identified in donkey fresh semen, in the presence or absence of oligomycin A (Omy A) or FCCP.

	SP1		SP2		SP3		SP4	
N	7,611		9,455		16,551		6,694	
Parameter	Mean ± SD	Q1, median, Q3	Mean ± SD	Q1, median, Q3	Mean ± SD	Q1, median, Q3	Mean ± SD	Q1, median, Q3
VCL μm/s)	168.4 ± 13.6	153.1, 168.1, 182.0	114.2 ± 16.1	98.0, 116.4, 132.8	70.2 ± 14.9	54.5, 68.7, 86.3	151.4 ± 19.5	132.3, 155.7, 173.9
VSL μm/s)	132.9 ± 18.0	113.5, 132.9, 150.9	101.7 ± 15.6	84.6, 102.3, 119.7	41.8 ± 9.3	30.5, 43.1, 53.8	54.5 ± 17.8	35.7, 56.0, 74.2
VAP μm/s)	152.4 ± 14.9	137.0, 151.8, 166.8	109.2 ± 16.1	92.1, 111.0, 128.2	47.4 ± 7.6	36.2, 47.8, 58.4	115.0 ± 19.1	98.9, 117.8, 134.2
LIN (%)	78.9 ± 9.2	69.8, 80.7, 88.7	89.1 ± 5.7	85.4, 91.3, 94.7	59.4 ± 13.2	49.8, 61.3, 71.2	35.9 ± 12.1	24.3, 37.4, 48.2
STR (%)	87.1 ± 7.2	81.8, 88.7, 94.4	93.3 ± 4.2	90.5, 95.5, 97.9	86.5 ± 12.2	84.9, 92.6, 95.5	47.3 ± 16.5	33.3, 49.2, 61.8
WOB (%)	90.3 ± 4.3	84.6, 91.1, 95.0	95.1 ± 3.6	92.4, 96.4, 98.8	68.1 ± 10.5	59.5, 68.5, 73.3	75.9 ± 9.1	69.3, 77.9, 84.2
ALH (μm)	3.7 ± 0.6	3.1, 3.6, 4.2	2.1 ± 0.5	1.6, 2.0, 2.5	2.6 ± 0.5	2.1, 2.5, 3.0	4.6 ± 1.1	3.7, 4.6, 5.5
BCF (Hz)	10.9 ± 2.5	9.0, 11.0, 13.0	8.2 ± 2.0	6.8, 8.0, 10.0	11.6 ± 3.1	9.0, 12.0, 14.0	8.0 ± 2.4	6.0, 8.0, 10.0

**These data were obtained after classifying sperm cells into motile subpopulations through principal component and cluster analyses.**

### Effects of Red Light Stimulation on Mitochondrial Membrane Potential in the Presence or Absence of Either Omy A or FCCP

**FIGURE 4:**
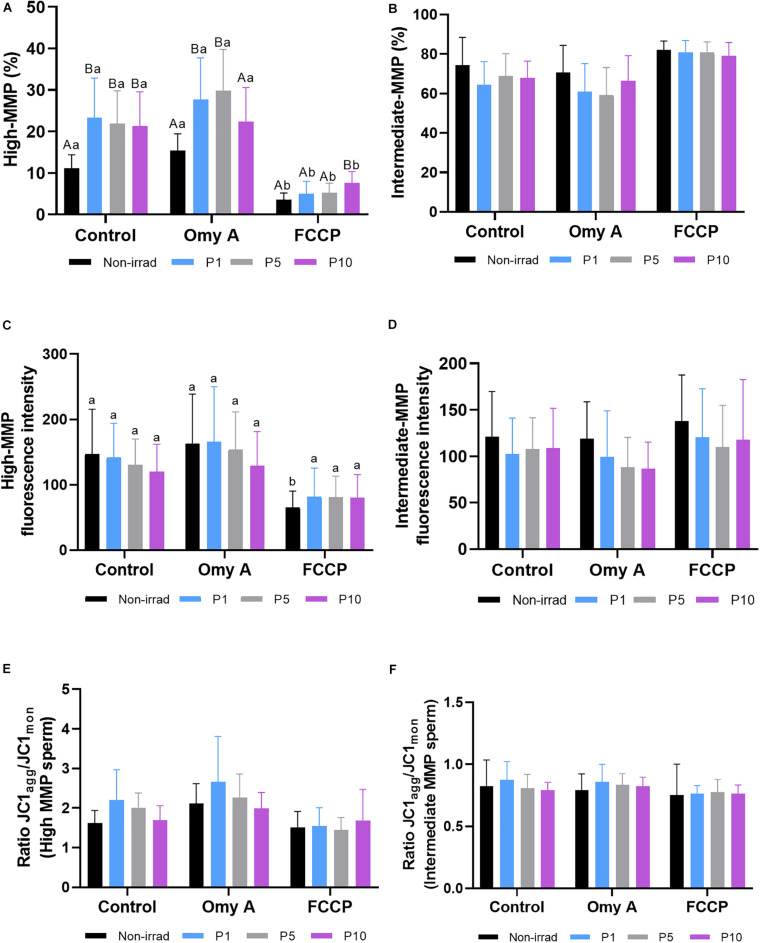
Effects of light stimulation on the mitochondrial membrane potential in the presence/absence of oligomycin A (Omy A) or FCCP. The results are presented as percentages of sperm with high mitochondrial membrane potential (MMP; JC1_a__gg_^++^; **(A)** and with intermediate mitochondrial membrane potential (MMP; JC1_a__gg_^+^; **(B)**; geometric mean of the JC1_a__gg_ fluorescence intensity (GMFI, FL2) in the sperm populations with high **(C)** and intermediate **(D)** MMP; and the JC1_a__gg_/JC1_m__on_ (GMFI FL2/GMFI FL1) ratios in sperm populations with high **(E)** and intermediate **(F)** MMP in control and irradiation patterns (P1, P5, or P10), in the presence/absence of Omy A. Different letters (*A, B*) indicate significant differences (*P* < 0.05) between non-irradiated samples and the different light-stimulation patterns used in the presence or absence of Omy A or FCCP. Different letters (*a, b*) indicate significant differences (*P* < 0.05) between Omy A or FCCP with respect to the treatment without the two disruptors for a given protocol (i.e., control, P1, P5, or P10). Data are shown as mean ± SD from eight separate experiments.

As shown in Figure 4A, irradiation for 1, 5, and 10 min significantly (*P* < 0.05) increased the percentages of spermatozoa with high MMP in samples without Omy A or FCCP. In treatments containing Omy A, irradiation for 1 min or 5 min significantly (*P* < 0.05) augmented the percentages of spermatozoa with high MMP compared to non-irradiated samples. Furthermore, percentages of spermatozoa with high MMP in samples containing FCCP were significantly (*P* < 0.05) higher in samples irradiated for 10 min than in their non-irradiated counterparts. In addition to this, percentages of spermatozoa with high MMP in non-irradiated samples and samples irradiated for 1, 5, or 10 min were significantly lower (*P* < 0.05) when FCCP was present than in the absence of this disruptor (Supplementary Figure 1).

As shown in Figure 4B, no significant differences in the percentages of sperm with intermediate MMP were observed between non-irradiated and irradiated samples, either in the presence or absence of Omy A and FCCP. In addition, no significant differences in the geometric mean of JC1_a__gg_ intensity (orange, FL2) of sperm populations with high (Figure 4C) and intermediate MMP (Figure 4D) were observed between irradiated and non-irradiated samples, either in the presence or absence of Omy A or FCCP. However, geometric mean of the intensity of JC1_a__gg_ (orange, FL2) in the sperm population with high MMP was significantly (*P* < 0.05) lower in the non-irradiated sample containing FCCP than in that without any inhibitor/disruptor (Figure 4C).

Finally, we also evaluated JC1_a__gg_/JC1_m__on_ ratios of sperm populations with high (Figure 4E) and intermediate MMP (Figure 4F). No significant differences between non-irradiated and irradiated samples were observed, either in the presence/absence of Omy A/FCCP, or within the same irradiation pattern comparing samples with and without disruptors.

### Effects of Red Light Stimulation on Intracellular ROS Levels in the Presence or Absence of Either Omy A or FCCP

**FIGURE 5:**
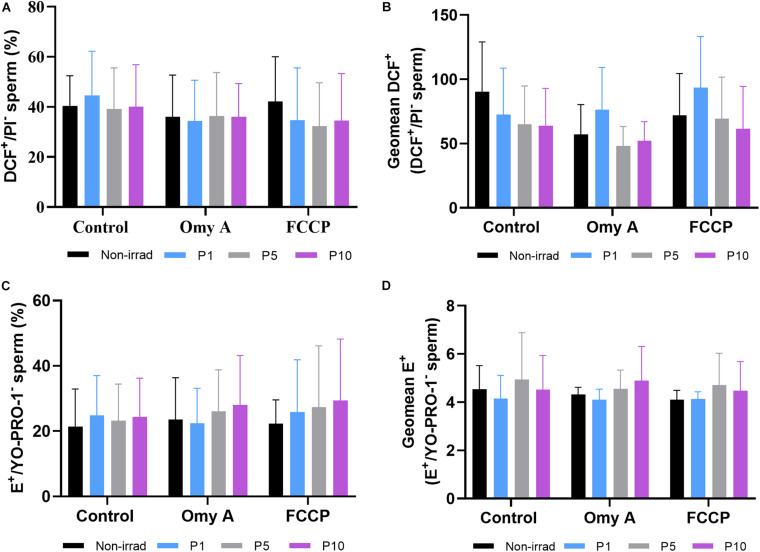
Effects of light-stimulation on the percentages of viable spermatozoa with high peroxide levels (DCF^+^/PI^–^; **(A)** geometric mean of DCF^+^ -intensity (GMFI, FL1 channel) in the population of viable spermatozoa with high peroxide levels **(B)** and percentages of viable spermatozoa with high superoxide levels (E^+^/YO-PRO-1^–^; **(C)** and geometric mean of E^+^ -intensity (GMFI, FL3 channel) in the population of viable spermatozoa with high superoxide levels **(D)** in the control and irradiated samples in the presence or absence of oligomycin A (Omy A) or FCCP. No significant differences were observed between non-irradiated sperm and the different light-stimulation patterns used in the presence/absence of oligomycin A or FCCP, nor between the results of the samples in the presence of Omy A or FCCP with respect to the treatment without the two disruptors for a given protocol (i.e., control, P1, P5, or P10). Data are shown as mean ± SD from eight separate experiments.

Figures 5A,B show the proportions of viable spermatozoa with high intracellular levels of peroxides (% DCF^+^/PI^–^ spermatozoa) and the GMFI of DCF^+^ in the population of viable sperm with high levels of peroxides (DCF^+^/PI^–^). No significant differences between irradiated and non-irradiated samples were observed, either in the presence or absence of Omy A or FCCP. In addition, no significant differences were found within each irradiation pattern when samples with and without disruptors (Omy A and FCCP) were compared.

Percentages of viable sperm with high levels of superoxides (% E^+^/YO-PRO-1^–^ spermatozoa; Figure 5C) and GMFI of E^+^ in the viable sperm population with high levels of superoxides (E^+^/YO-PRO-1^–^; Figure 5D) did not significantly differ between irradiated and non-irradiated samples, or between treatments with and without disruptors (Omy A and FCCP) within each irradiation pattern.

### Effects of Red Light Stimulation on Intracellular Calcium Levels in the Presence or Absence of Either Omy A or FCCP

**FIGURE 6:**
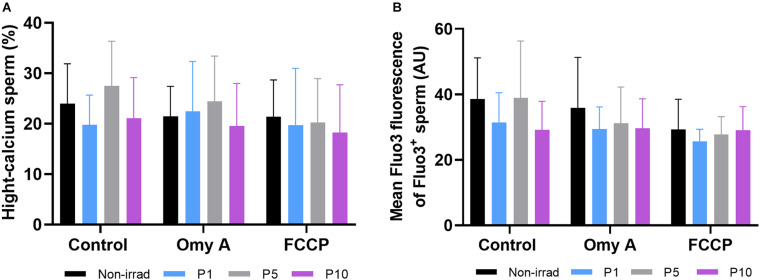
Effects of light-stimulation on percentages of spermatozoa with high intracellular calcium levels (Fluo3^+^); **(A)** and geometric mean intensity of Fluo3^+^ **(B)** in the presence/absence of either oligomycin A or FCCP. No significant differences were observed between non-irradiated sperm and the different light stimulation patterns used in the presence or absence of oligomycin A (Omy A) or FCCP, nor between the results of the samples in the presence of Omy A or FCCP with respect to the treatment without the two inhibitors for a given protocol (i.e., control, P1, P5, or P10). Data are shown as mean ± SD from eight separate experiments.

Percentages of viable sperm with high intracellular calcium levels did not differ between irradiated and non-irradiated samples in the presence/absence of Omy A and FCCP, nor between the presence/absence of disruptors within each irradiation pattern (Figure 6A). Similar results were obtained in the case for the GMFI of Fluo3^+^ in the viable sperm population with high intracellular calcium levels (Fluo3^+^/PI^–^; Figure 6B).

### Effects of Red Light Stimulation on Intracellular Levels of ATP and Oxygen Consumption in the Presence or Absence of Either Omy A or FCCP

**FIGURE 7:**
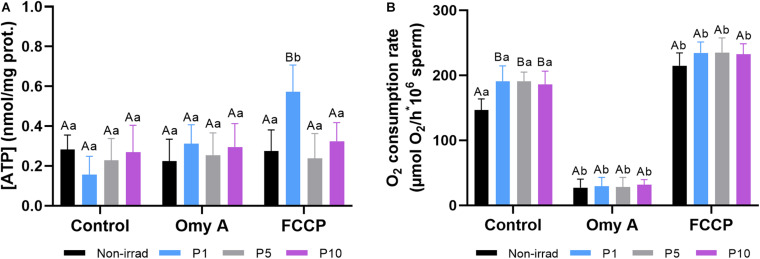
Effects of light-stimulation on intracellular ATP levels **(A)** and O_2_ consumption rate **(B)** in the presence/absence of either oligomycin A (Omy A) or FCCP. Different letters (*A, B*) indicate significant differences (*P* < 0.05) between non-irradiated sperm and the different light stimulation patterns used in the presence or absence of Omy A or FCCP. Different letters (*a, b*) indicate significant differences (*P* < 0.05) between Omy A or FCCP with respect to the treatment without the two disruptors for a given protocol (i.e., control, P1, P5, or P10). Data are shown as mean ± SD from eight separate experiments.

Figure 7A shows the intracellular levels of ATP observed in non-irradiated and irradiated samples in the presence or absence of Omy A or FCCP. No significant differences were observed between non-irradiated and irradiated samples were observed, either in the presence or absence of oligomycin A. In the presence of FCCP, intracellular ATP levels were significantly (*P* < 0.05) higher in sperm irradiated for 1 min than in those non-irradiated. In addition, samples irradiated for 1 min showed significantly (*P* < 0.05) higher intracellular levels of ATP in the presence than in the absence of FCCP.

Figure 7B shows that light stimulation (*P* < 0.05) significantly increased the O_2_ consumption rate in all three protocols (i.e., P1, P5, and P10) compared to the non-irradiated control. In samples containing Omy A, no significant differences between non-irradiated and irradiated samples were observed. However, O_2_ consumption rates in non-irradiated and irradiated samples containing Omy A were significantly (*P* < 0.05) lower than in the ones that did not contain this inhibitor. On the other hand, no significant differences between irradiated and non-irradiated samples were observed in samples containing FCCP. However, samples containing FCCP showed significantly (*P* < 0.05) higher O_2_ consumption rates than those without this disruptor, regardless of whether they were irradiated.

### Effects of Red Light Stimulation on Cytochrome C Oxidase Activity in the Presence or Absence of Either Omy A or FCCP

**FIGURE 8:**
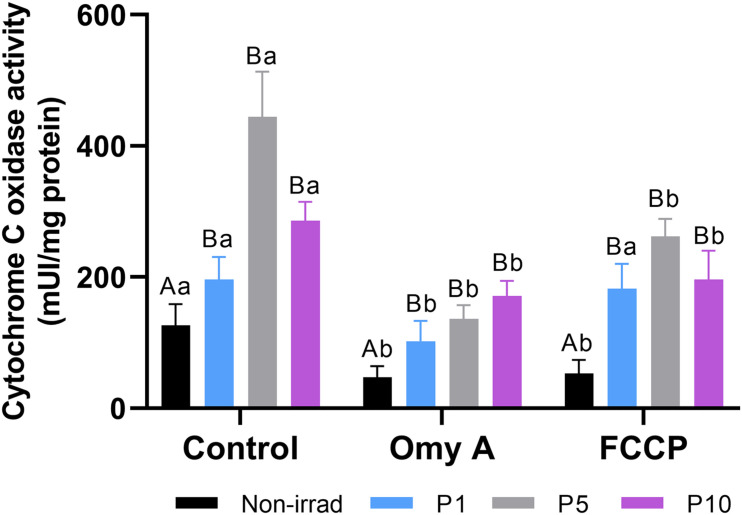
Effects of light-stimulation on cytochrome C oxidase activity in the presence/absence of either oligomycin A (Omy A) or FCCP. Different letters (*A, B*) indicate significant differences (*P* < 0.05) between non-irradiated sperm and the different light stimulation patterns used in the presence or absence of Omy A or FCCP. Different letters (*a, b*) indicate significant differences (*P* < 0.05) between Omy A or FCCP with respect to the treatment without the two inhibitors for a given protocol (i.e., control, P1, P5, or P10). Data are shown as mean ± SD from eight separate experiments.

As shown in Figure 8, irradiation for 1, 5, or 10 min induced a significant increase (*P* < 0.05) in CCO activity compared to non-irradiated samples. Although these effects were observed in both the presence and absence of Omy A or FCCP, the highest CCO activity was observed in sperm irradiated for 5 min in the absence of Omy A/FCCP.

While neither Omy A nor FCCP affected the significant (*P* < 0.05) increase in CCO activity observed in irradiated sperm compared to non-irradiated sperm, samples containing Omy A or FCCP showed significantly (*P* < 0.05) lower CCO activity than when these two molecules were absent from non-irradiated sperm and sperm irradiated for 5 min and 10 min. In contrast, CCO activity in sperm irradiated for 1 min was significantly (*P* < 0.05) lower when Omy A was present than when this inhibitor was absent.

## Discussion

The results obtained in the present study with regard to the potential of mitochondrial membrane, sperm kinetic parameters, the structure of motile sperm subpopulations, CCO activity and oxygen consumption rate support that irradiation with red LED light affects mitochondrial activity of donkey sperm. Furthermore, the impact of red light has been found to rely upon the time of exposure (i.e., 1, 5, or 10 min), regardless of the presence of Omy A and FCCP, which is in agreement with previous studies conducted in other mammalian species, such as pigs, dogs, buffalos, humans, donkeys and horses (Corral-Baqués et al., 2009; Abdel-Salam et al., 2011; Salman Yazdi et al., 2014; Yeste et al., 2016; Catalán et al., 2020a,b,c). In this context, it is worth emphasizing that a high individual variation was observed, which indicates that apart from the relevance of the stimulation pattern, time and intensity (Yeste et al., 2016), the sperm response to red light also depend on the functional status of the cell (Catalán et al., 2020a). Although the mechanisms underlying this different response have yet to be elucidated, this work suggests that, in donkey sperm, red light acts on mitochondrial photosensitizers and that the energy supplied to the mitochondrial electron chain following irradiation is related to the time of exposure and intensity. Therefore, the exact level of energy provided to sperm through red light stimulation and the overall sperm function status appear to be on the basis of the different impact observed between treatments and species.

With regard to the effects of red light on the mitochondrial electron chain observed in this work, it is reasonable to surmise that this could be explained by the direct action of red light on mitochondrial photosensitizers (reviewed in Yeste et al., 2018). Indeed, we observed that red light affected the activity of CCO, which is a crucial component of the mitochondrial electron chain and is sensitive to light at a wavelength ranging from 630 to 660 nm (Lynch and Copeland, 1992). These observed increases in CCO activity are similar to those reported in ram semen irradiated with He-Ne laser (632.8 nm; Iaffaldano et al., 2016) and in liquid-stored pig semen irradiated with LED (Blanco-Prieto et al., personal communication), which would also support the hypothesis that mitochondrial photosensitizers play a relevant role in the effects of red light upon sperm cells. On the other hand, our results showed that neither irradiation nor the presence of Omy A or FCCP had any detrimental effect on sperm viability, which was evaluated through the SYBR14^+^/PI^–^ test. These results are in agreement with Yeste et al. (2016) and Pezo et al. (2019), who conducted their studies in liquid-stored pig semen; Catalán et al. (2020a), who evaluated the effects of red light irradiation on fresh and cooled-stored donkey semen; and Catalán et al. (2020b, c) that used frozen-thawed and fresh horse sperm.

As far as the effects of red light on sperm motility are concerned, it is worth mentioning that while no significant differences were observed in the percentages of TMOT and PMOT between non-irradiated and samples irradiated with red light, light-stimulation for 5 min or 10 min in the absence of Omy A/FCCP significantly increased VAP, which is in agreement with previous studies conducted in dogs (Corral-Baqués et al., 2005, 2009), cattle (Siqueira et al., 2016), buffalos (Abdel-Salam et al., 2011), pigs (Yeste et al., 2016), donkeys (Catalán et al., 2020a), and horses (Catalán et al., 2020b, c). We also evaluated the effects of red light on motile sperm subpopulations and we identified four different subpopulations, which is in agreement with that previously reported in the donkey (Miró et al., 2005). Remarkably, we observed that irradiation for 1 min increased the percentages of sperm belonging to SP1, which was the one that included the fastest and most linear motile sperm. Thus, our data suggest that irradiation of fresh donkey sperm with red light modifies the structure of motile sperm subpopulations by increasing the proportions of faster and more linear sperm cells. In addition, our results concur with a previous study conducted on dog semen, in which stimulation with red light (laser) significantly increased the proportions of the fastest sperm subpopulation (Corral-Baqués et al., 2009) and another one carried out with fresh horse sperm where, in a similar fashion to this work, an increase in the most rapid and linear subpopulation was observed (Catalán et al., 2020c). These changes in the characteristics of the motile sperm subpopulations, together with those observed in sperm kinetic parameters, indicate that not only does irradiation with red light increase the speed but also alters the motility pattern of donkey sperm. While there is not, at present, a clear explanation about how red light stimulation affects sperm motility, the aforementioned impact on mitochondrial function could provide some clues. Related to this, it is worth mentioning that the increase observed in VAP and in the proportions of spermatozoa belonging to SP1 was concomitant with a rise in the percentages of sperm with high MMP, determined through JC1. These findings agree with Siqueira et al. (2016), who found that irradiation with a He-Ne laser at a wavelength of 633 nm augmented the percentage of sperm with intermediate MMP. Similar results were found in pig, donkey and horse sperm, since irradiation with red LED light at a wavelength between 620 and 630 nm increased the percentages of sperm with high MMP (Yeste et al., 2016; Catalán et al., 2020a,b,c). Therefore, the current study suggests that stimulation with red light increases mitochondrial activity through endogenous photosensitizers, such as cytochrome C (Begum et al., 2013; Iaffaldano et al., 2016; Yeste et al., 2016; Catalán et al., 2020a,b,c), which could, in turn, lead to greater motility of sperm and higher fertilization potential (Breitbart et al., 1996).

Recent studies indicate that oxygen consumption represents an alternative measure of mitochondrial activity, which could be better than the use MMP markers, such as JC1 (Moscatelli et al., 2017; Meyers et al., 2019). Oxygen consumption rate would also provide an indirect measure of ATP produced by oxidative phosphorylation in mammalian sperm (Meyers et al., 2019). The results obtained in this study showed an increase in oxygen consumption on all irradiated samples (namely, light-stimulated for 1, 5, or 10 min) in the absence of Omy A or FCCP compared to the non-irradiated control. While these results are in agreement with those obtained from the evaluation of some motility parameters, the structure of motile subpopulations, the percentages of viable sperm with high MMP and the activity of cytochrome C oxidase, it is surprising that no link to intracellular levels of ATP was observed, since no differences in this parameter were found when non-irradiated and irradiated samples without oligomycin A/FCCP were compared. Moreover, no relationship between intracellular ATP levels and other mitochondrial parameters was observed in samples irradiated for 1 min in the presence of FCCP. The increase in the potential of the mitochondrial membrane is associated with changes in the consumption of ATP and in the activity of respiratory chain enzymes (Alberts et al., 2002; Zorova et al., 2018). Related with this, Iaffaldano et al. (2016) observed that light stimulation of frozen-thawed ram sperm with a He-Ne laser increased ATP content and the activity and affinity of cytochrome C oxidase for its substrate (cytochrome C). Interestingly, these authors found that CCO activity and ATP content were positively correlated with each other and with sperm motility, supporting the hypothesis that the effects of red light upon sperm are mediated by mitochondria. However, and as aforementioned, we did not observe that correspondence in the case of donkey sperm. An explanation about why irradiation did not result in a clear increase of intracellular ATP levels could be that ATP in sperm is mainly synthesized through two routes, glycolysis and mitochondrial oxidative phosphorylation, without dismissing other energy sources available to sperm such as β-oxidation (Storey and Keyhani, 1974; Amaral et al., 2013). The precise balance of ATP production from both sources is very dynamic and multifactorial, undergoing rapid changes not only between species, but also within the same cell under different physiological and environmental conditions. The intracellular changes that are related to the complex relationship between mitochondrial oxidative phosphorylation and glycolysis, and that could explain these effects will be later discussed in more detail.

In addition to the aforementioned, the electron chain is known to play a fundamental role in the generation of reactive oxygen species (ROS), since mitochondria are the most important source of ROS generation in eukaryotic cells (Zhao et al., 2019). A previously established hypothesis indicates that light-stimulation increases ROS production by sperm (Cohen et al., 1998; Zan-Bar et al., 2005). Furthermore, cytochrome complexes are also involved in the intrinsic apoptotic pathway (Cai et al., 1998), and it has been surmised that both generation of ROS and modulation of apoptotic-like changes could be crucial to elicit and modulate the achievement of capacitated status by sperm (Ortega-Ferrusola et al., 2009; Aitken et al., 2015). Against this background, one could reasonably suggest that the red light-induced changes in CCO activity observed herein could ultimately affect the lifespan and capacitation status of mammalian sperm. However, our results showed that light-stimulation of fresh donkey sperm did not increase intracellular levels of ROS or calcium, which is a crucial secondary messenger involved in early and late capacitation events (Yeste, 2013; Correia et al., 2015). Thus, our data differ from those observed in donkeys and other species in which irradiation was found to increase intracellular ROS (Cohen et al., 1998; Zan-Bar et al., 2005; Catalán et al., 2020a) and intracellular calcium levels (Lubart et al., 1992; Cohen et al., 1998). These differences could be due to the high individual variability in the sperm response to red light, as well as to differences in the light source, intensity and irradiation pattern, as previous reports found for intracellular levels of ROS (Catalán et al., 2020a) and calcium (Breitbart et al., 1996). Moreover, the existence of a complex homeostasis system aimed at maintaining ROS levels within a physiological range, which would include systems such as the glutathione peroxidase-glutathione reductase complex (GPX/GSR) and enzymes such as intracellular peroxidases (see Peña et al., 2019 for review), would allow sperm to maintain ROS levels in the event of a temporarily induced stress like the one caused by light-stimulation. Nevertheless, further research should be conducted to confirm this hypothesis.

Regarding our results obtained in samples containing Omy A or FCCP, one should note that irradiation in the presence of these two molecules increased the percentages of sperm with high MMP, compared to the non-irradiated control in the presence of these molecules. Furthermore, non-irradiated samples in the presence of FCCP exhibited lower intensity of JC1_a__gg_ fluorescence in the sperm population with high MMP, which did not occur in irradiated samples. These findings also support that light-stimulation of sperm exerts its effects via MMP. At this point, it is worth mentioning that some studies have recently questioned the use of MMP probes to assess the potential of the mitochondrial membrane due to its non-specific nature (Meyers et al., 2019). These reports point up that MMP probes are cationic and their accumulation rate inside the mitochondria is inversely proportional to the potential of the internal mitochondrial membrane; hence, MMP probes like JC1, which was the one used in this study, may not be reliable as a quantitative measure of MMP without adequate controls. Therefore, these previous studies suggest that high/low MMP controls, such as ATP synthase inhibitors like Omy A and MMP uncouplers like FCCP or DNP, are needed (Meyers et al., 2019). Remarkably, the results obtained in this work with Omy A/FCCP showed this controlling effect, since percentages of sperm with high MMP in samples without disruptors were lower than those containing Omy A, and significantly higher than those containing FCCP. Regarding sperm motility parameters in the presence of these two molecules, they were similar to those found by Ramió-Lluch et al. (2014) and Nesci et al. (2020) in previous studies conducted with pig semen. According to Nesci et al. (2020), the reduced motility observed in treatments containing Omy A or FCCP could result from the decrease in ATP content caused by these two molecules. However, our results indicate that the presence of Omy A and FCCP in irradiated and non-irradiated sperm reduces sperm motility without causing a decrease on overall sperm ATP levels. These findings observed in the presence of Omy A are similar to those seen by Ramió-Lluch et al. (2014), who reported that the control exerted by Omy A-sensitive ATP synthase over pig sperm motility does not seem to be related to its inhibiting effect upon ATP levels. In this context, it is worth mentioning that a previous study reported that mitochondrial respiration of pig sperm incubated in the presence of glucose only contributes to 5% of the total energy produced by the cell, the other 95% being obtained from glycolysis (Marín et al., 2003). Therefore, it is surprising that the relatively low levels of energy produced through ATP synthase affected sperm motility as much as they did in the study of Ramió-Lluch et al. (2014), especially if one bears in mind that these authors did not observe an alteration of overall sperm energy levels. Related with this, in a recent study, Nesci et al. (2020) observed that, despite pig sperm being regarded to rely upon glycolysis, the motility of these cells is highly dependent on the ATP produced through mitochondrial oxidative phosphorylation. In other studies, such as those carried out by Iaffaldano et al. (2016) on ram sperm and Odet et al. (2013) on mouse sperm, an association between overall ATP content and sperm motility has also been observed. In the case of mice, one should keep in mind that their sperm have been reported to maintain their function through the ATP originated from glycolysis or mitochondrial respiration indistinctly (Pasupuleti, 2007). In addition to this, a recent study published by Balbach et al. (2020) has demonstrated a functional link between these two pathways during mouse sperm capacitation. All these data strongly point to the existence of species-specific differences on the mechanisms by which mammalian spermatozoa produce ATP via glycolysis or oxidative phosphorylation (Rodríguez-Gil and Bonet, 2016). Our results suggest that changes in intracellular ATP levels cannot be taken as a direct indicator of changes affecting mitochondrial function, since glycolysis and probably other metabolic pathways present in sperm also produce ATP; thus, further metabolomics studies in donkey sperm are needed to understand the precise glycolysis/oxidative phosphorylation balance in these cells.

While the evidence reported in this and another study in pigs (Blanco-Prieto et al., personal communication) indicates that the effects of red light on sperm would be related to the direct effect on intracellular light-sensitive proteins, especially the impact on endogenous photosensitizers, such as mitochondrial cytochromes, other pathways or factors that might modulate the effects of red light on mammalian sperm could also be involved (reviewed in Yeste et al., 2018). These pathways or factors include light-sensitive receptors, such as opsins (Pérez-Cerezales et al., 2015) and transient receptor potential proteins (TRP) (De Blas et al., 2009; Bahat and Eisenbach, 2010; Gibbs et al., 2011), whose one of their most probable functions in mammalian sperm is the regulation of thermotaxis (Wu et al., 2010; Pérez-Cerezales et al., 2015). Thermotaxis could be, in fact, an important modulator of light stimulation, since mammalian sperm are sensitive to temperature changes as small as 0.0006°C (Bahat et al., 2012; Pérez-Cerezales et al., 2015). Therefore, it is reasonable to suggest that red light stimulation could also act through this pathway, especially if one takes into consideration that, in this study, the effects of irradiation on MMP and ATP content, which rely on glycolysis/oxidative phosphorylation balance, differed. In addition, one should not discard that signaling transduction pathways triggered when opsins and TRP receptors are activated could change ATP production via glycolysis as well as the balance between glycolysis and oxidative phosphorylation.

## Conclusion

In conclusion, our results indicate that the effects induced by the stimulation of fresh donkey sperm with red LED light are related to mitochondrial photosensitizers, such as CCO, which modify the activity of the mitochondrial electron chain; the effect of red light on these photosensitizers depends on the time of exposure, among other factors. However, these findings do not exclude that this mitochondrial mechanism could work in conjunction with other pathways, such as thermotaxis, via plasma membrane receptors.

## Data Availability Statement

The raw data supporting the conclusions of this article will be made available by the authors, without undue reservation, to any qualified researcher.

## Ethics Statement

All jackasses used in this study were semen donors and no manipulation to animals, apart from semen collection in the authorized center, was made. The animal study was reviewed and approved by the Ethics Committee for Animal and Human Experimentation (CEEAH) of our institution (Autonomous University of Barcelona; authorization code: CEEAH 1424.

## Author Contributions

MY, JR-G, and JM: conceptualization. JC, MP, LT-R, OB-P, JM, MY, and JR-G: methodology and investigation. JM, MY, and JR-G: validation, project administration, and supervision. JR-G: formal analysis and data curation. JC: writing—original draft preparation. JM and MY: writing—review and editing, funding acquisition, and resources. All authors have read and agreed to the published version of the manuscript.

## Conflict of Interest

JR-G and MY were inventors of a patent entitled ‘Method and apparatus for improving the quality of mammalian sperm’ (European Patent Office, No. 16199093.2; EP-3-323-289-A1), which is owned by Instruments Utils de Laboratori Geniul, SL (Barcelona, Spain). The remaining authors declare that the research was conducted in the absence of any commercial or financial relationships that could be construed as a potential conflict of interest.

## Supplementary Material

Supplementary Figure 1 Representative flow-cytometry dot-plots observed in non-irradiated samples and samples irradiated for 5 min, in the presence/absence of FCCP and Omy A.
