# Supplementary figures and images for: Red LED Light Acts on the Mitochondrial Electron Chain of Donkey Sperm and Its Effects Depend on the Time of Exposure to Light
Source: Front Cell Dev Biol. 2020 Dec 7;8:588621. doi: 10.3389/fcell.2020.588621 (PMC7750462; doi:10.3389/fcell.2020.588621)

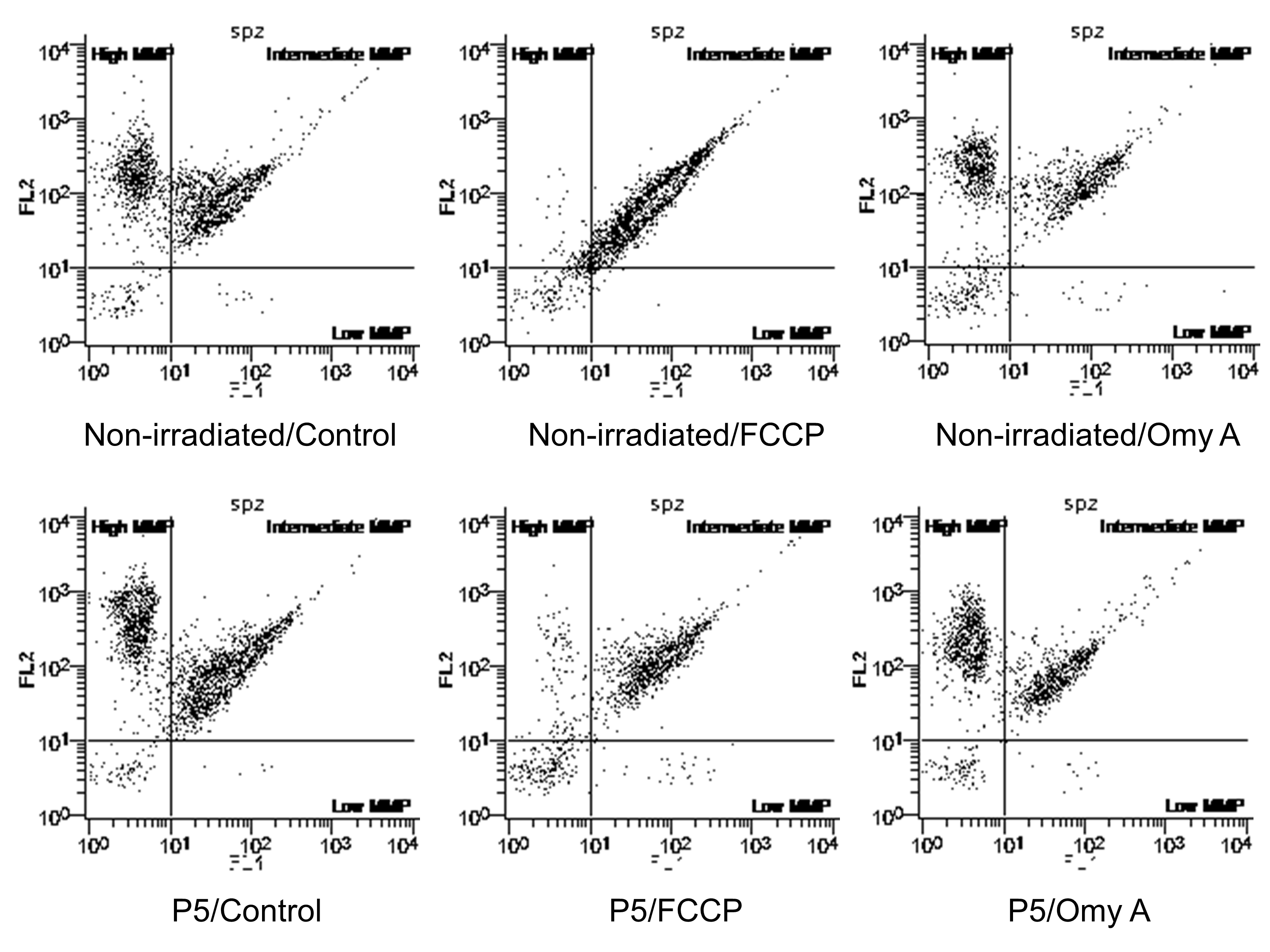

Supplement: Supplementary Figure 1 — Representative flow-cytometry dot-plots observed in non-irradiated samples and samples irradiated for 5 min, in the presence/absence of FCCP and Omy A. [file Image_1.TIF]
